# Anticancer Effects of Withanolides: In Silico Prediction of Pharmacological Properties

**DOI:** 10.3390/molecules30112457

**Published:** 2025-06-04

**Authors:** Gustavo Werneck de Souza e Silva, André Mesquita Marques, André Luiz Franco Sampaio

**Affiliations:** 1 Laboratório de Farmacologia Molecular, Farmanguinhos, Fundação Oswaldo Cruz (FIOCRUZ), Rua Sizenando Nabuco 100, Manguinhos, Rio de Janeiro 21041-250, Brazil; gustavo.werneck@fiocruz.br; 2 Laboratório de Tecnologia Para Biodiversidade em Saúde/TecBio, Farmanguinhos, Fundação Oswaldo Cruz (FIOCRUZ), Rua Sizenando Nabuco 100, Manguinhos, Rio de Janeiro 21041-250, Brazil; andre.marques@fiocruz.br

**Keywords:** withanolides, ADMET, withaferin, drug development, cancer, natural products

## Abstract

Withanolides are a class of naturally occurring C-28 ergostane steroidal lactones with an abundance of biological activities, and their members are promising candidates for antineoplastic drug development. The ADMET properties of withanolides are still largely unknown, and in silico predictions can play a crucial role highlighting these characteristics for drug development, shortening time and resources spent on the development of a drug lead. In this work, ADMET properties of promising antitumoral withanolides were assessed. Each chemical structure was submitted to the prediction tools: SwissADME, pkCSM–pharmacokinetics, admetSAR v2.0, and Molinspiration Cheminformatics. The results indicate a good gastrointestinal absorption rate, inability to cross the blood–brain barrier, CYP3A4 metabolization, without inhibition of other P450 cytochromes, high interaction with nuclear receptors, and a low toxicity. It was also predicted for the inhibition of pharmacokinetics transporters and some ecotoxicity. This demonstrates a viability for oral drug development, with low probabilities of side effects.

## 1. Introduction

Cancer is a worldwide health challenge that affects millions of patients every year. Environmental and genetic factors both increase the risk of developing cancer, compromising life expectancy and the patients’ quality of life [1]. The adverse effects of anticancer drugs can be a major factor of the impairment of the patients’ quality of life, where some of these effects can be irreversible [2]. The complexity and heterogeneity of cancer demand innovative and multifaceted approaches to combat its relentless progression [3]. Thus, in the last decades, despite the availability of several approved anticancer drugs, the ongoing research and development of new antitumoral agents is always a constant need as a foremost public health demand [4].

Naturally occurring plant secondary metabolites have been successfully used as natural-based templates by offering some structural advantages, in comparison with conventional synthetic molecules as drug leads. Regarding medicinal plants, their use in traditional medicine may provide some insights regarding efficacy and safety [5]. The interest in natural products as potential sources for drug discovery lies not only in their proven track record as therapeutics, but in their inherent chemical diversity. The metabolites complex scaffold structures and possible multiple chiral centers were fine-tuned over millions of years of evolution, that challenge traditional drug design paradigms and are the result of an interplay between living organisms and their environments [6]. These natural compounds have demonstrated a valuable ability to interact with a wide range of biological pathways involved in human health and disease, often acting by multiple modes of action and an inherent ability to engage multiple cellular targets simultaneously. This capacity makes them valuable assets in combating drug-resistant diseases with complex etiologies, including those involved in cancer initiation, cell proliferation, angiogenesis, and metastasis [7].

Historically, natural products were a major contributor to the development of new drugs, with more than 50% of anticancer drugs relating to this source [8]. The evolution of cancer drug discovery has witnessed phases of intense focus on synthetic compounds, which have temporarily overshadowed the exploration of natural products. However, in the wake of persistent challenges posed by drug resistance, systemic toxicity, and limited efficacy of conventional therapies, the scientific community has rediscovered the value of nature’s pharmacopeia [9]. The potential for natural products to modulate key signaling cascades and interact with specific molecular targets has been validated in preclinical studies, paving the way for several promising candidates to enter clinical trials [10].

Withanolides are a class of naturally occurring C-28 ergostane steroidal lactones with more than 600 compounds isolated and characterized from various plant species, particularly from the Solanaceae family [11]. Primary pharmacological studies involving the withanolide class have shown a plethora of biological activities, including in vitro antineoplastic effects [12,13,14].

Recent investigations into the molecular mechanisms underlying withanolide-mediated anticancer effects have unveiled their ability to interfere with various signaling pathways crucial for cancer cell survival, proliferation, and metastasis [15,16]. The inhibition of key inflammatory signaling pathways, such as NF-κB, JAK/STAT, AP-1, PPARγ, Hsp90 Nrf2, and HIF-1, highlights their potential to attenuate inflammation-mediated chronic diseases, including cancer-promoting cascades, while inducing tumor cell apoptosis and cell cycle arrest [17,18]. Additionally, withanolides also have been shown to sensitize cancer cells to conventional chemotherapy and radiotherapy, potentially overcoming drug resistance and enhancing treatment outcomes [19].

In preclinical studies, withanolides have exhibited promising efficacy against a broad spectrum of cancers, including breast, prostate, lung, colon, pancreatic, and ovarian cancers, among others [20]. Their ability to selectively target cancer cells while sparing normal cells holds great promise in minimizing the adverse effects associated with conventional therapies. Moreover, their anti-metastatic properties and potential to inhibit angiogenesis may underpin their effects on cancer progression [21]. However, despite several pharmacological activities already reported, the ADMET properties of many withanolides are largely unknown. In this work, we have selected withanolides with antitumoral in vitro effects to predict in silico their toxicological effects, their safety for biological systems, their interactions with important proteins that affect drug pharmacokinetic, their metabolism, and whether or not they may grant tumor resistance to chemotherapy.

Pharmacological studies with withanolides have demonstrated anticancer properties on human cancer cells in vitro, as well as in vivo, inhibiting tumor growth and inducing metastasis suppression [22,23,24]. Recently, pharmacological investigations have suggested withanolides’ possible mechanisms of action, including production of ROS, cell cycle disturbance, and cytoskeleton alterations [25,26]. However, the precise molecular mechanism and receptor(s) involved in withanolides’ actions on cancer cells is still largely unknown.

In this context, in silico predictions may play a crucial role in streamlining the drug discovery process. In silico virtual screening techniques can efficiently assess the interactions between natural metabolites and target proteins, helping prioritize the most promising candidates for further experimental validation [27]. This approach significantly reduces the number of compounds that need to be synthesized, and tested in vitro or in vivo, prevents the duplication of efforts and enables researchers to focus on compounds with higher probabilities of activity on specific diseases. In silico studies also contribute for structure optimization for activity, selectivity, and improvement of pharmacological properties, reducing the time and resources spent on less promising leads [28].

The application of in silico predictions has become an important component of pharmaceutical research and development that can shorten and cheapen the process by using previous data of known molecules, and comparing them to new structures [29,30]. The use of computer modeling, to predict the absorption, distribution, metabolism, and excretion (ADME), to predict and understand structure–properties relationships of the pharmacokinetics and drug metabolism properties for novel compounds, is widely used in the development of new drug candidates [31]. A variety of computational techniques make it possible to predict the toxicity (T) of a given chemical compound and is one of the alternatives to animal testing, being widely used in design and drug discovery and development [32].

Despite the relevance of in silico predictions and their role in drug discovery and medicinal chemistry, it is important to note some limitations to the accuracy of prediction tools. Substances have a variety of characteristics that can interfere with pharmacological properties and biological responses depending on the organ, tissue, or cell type. Each predictive tool has its own database and training method (built based on experimental data), and the size and the quality of the data used for training can alter the results from its usage. Thus, it is important to use more than one prediction tool to evaluate ADMET characteristics for accurate prediction. The integration of in silico predictions and their confirmation with an experimental approach, can accelerate the identification of lead compounds and increase the efficiency of discovering novel drugs from natural sources [33].

## 2. Results

### 2.1. Withanolides Structures and ADMET Studies

Withanolides are steroidal molecules with 28 carbons, with a wide range of biological activities. These compounds are predominantly found in species of the Solanaceae family, their first member was isolated from *Withania somnifera* (Withaferin A) and are scarcely found in plants of other families.

**Figure 1:**
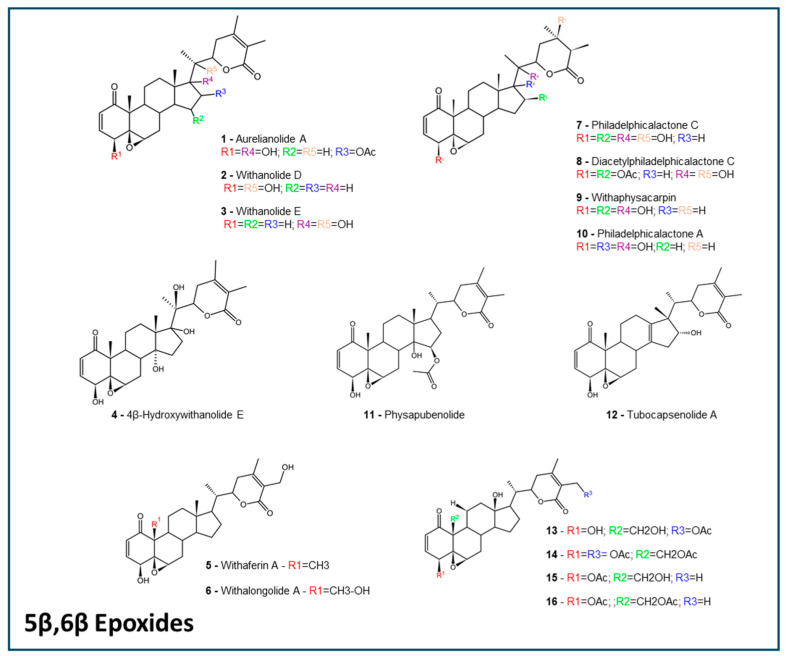
The 5β,6β epoxides withanolides. The structures were designed using the software ACD/ChemSketch 2020.2.0 (ACD/Labs).

**Figure 2:**
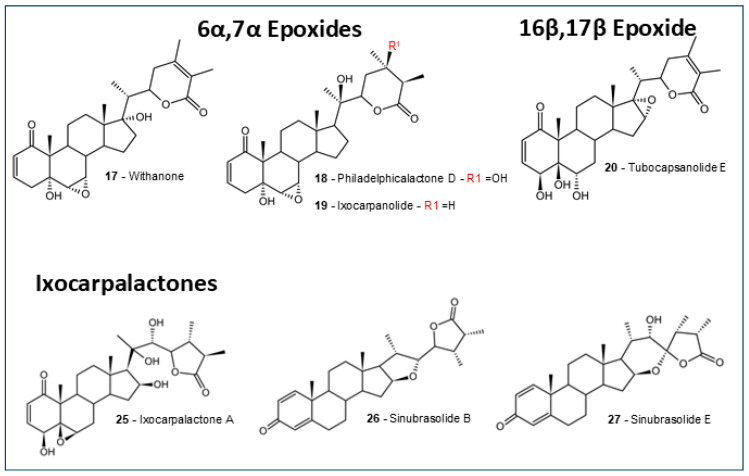
The 6α,7α epoxides, 16β,17β epoxides, and ixocarpalactones withanolides. The structures were designed using the software ACD/ChemSketch 2020.2.0 (ACD/Labs).

**Figure 3:**
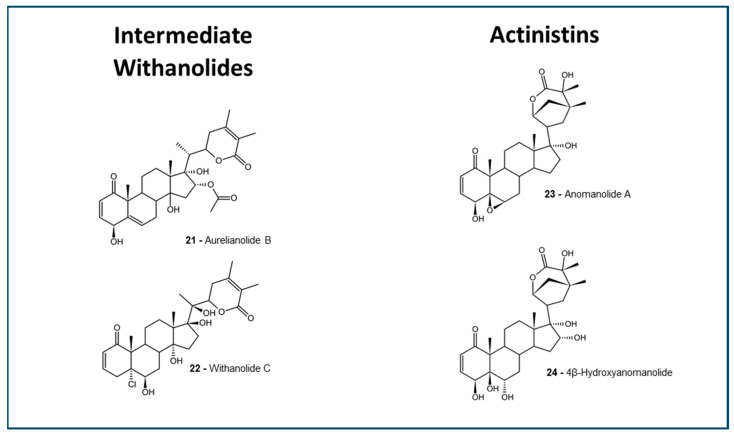
Actinistins and intermediate withanolides. The structures were designed using the software ACD/ChemSketch 2020.2.0 (ACD/Labs).

The selected withanolides (Figure 1, Figure 2 and Figure 3) are intended to represent a plethora of subtypes, to better understand the ADMET properties among the different structures. In the past years, in silico predictions, coupled with computational approaches, have played an important role in advancing the study of withanolides and their potential applications in drug discovery and development. Here, we present some key aspects of selected antitumoral withanolides for in silico ADMET predictions.

**Figure 4:**
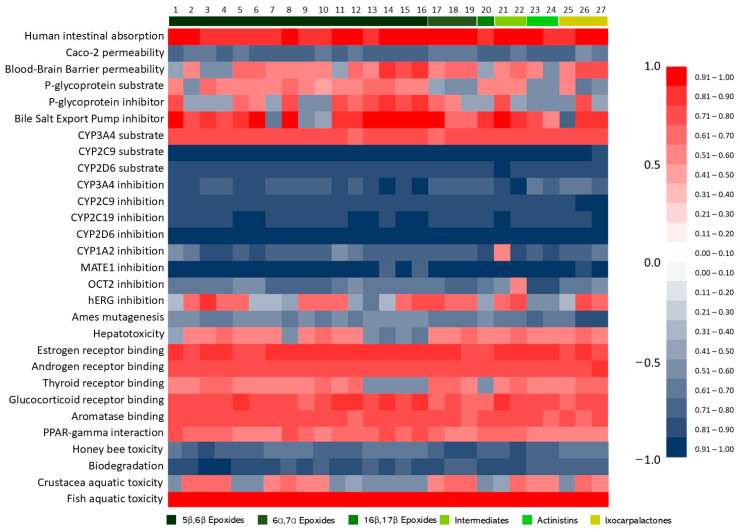
Heatmap constructed from admetSAR predictions. A total of 27 withanolides were assessed for their ADMET properties. Results range from 1.0 (+) to 1.0 (−), and were plotted following a color intensity scheme.

To better visualize the in silico ADMET predictions, data were expressed as a probability and organized in a heatmap (Figure 4) and tables for binary and other predictions. Data for each withanolide are available in the supplementary materials (Tables S3–S6).

### 2.2. Oral Absorption

#### 2.2.1. Lipinski’s Rule of Five

Lipinski’s rule of five is a set of guidelines used in medicinal chemistry and drug discovery to assess the likelihood of a compound’s oral bioavailability and permeability. In his work [34], Lipinski has categorized some desired drug properties as good absorption and permeation through the gastrointestinal tract. These properties, known as “Lipinski’s rule of 5” were described based on the characteristics of drugs in clinical use with the following criteria: molecular weight (MW) ≤ 500 Daltons; octanol–water partition coefficient (LogP) ≤ 5; hydrogen bond donors (HBD) ≤ 5; and hydrogen bond acceptors (HBA) ≤ 10. As shown in Table 1, of the 27 withanolides analyzed, 26 fit the rule of five, and could be predicted with good oral absorption and permeation. Among them, 13 withanolides violated no rule, and 13 withanolides violated one rule, and only 4β-Hydroxyanomanolide violated two rules.

Furthermore, the topological polar surface area (TPSA) obtained in Molinspiration can be used to predict the percentage of absorption, using the equation 109-[0,345xTPSA] [35]. The results, as shown in Table 1, indicate that the ixocarpanolides have the highest percentage of absorption, as both sinubrasolides assessed showed high individual values, with 90.8% and 83.9%, for Sinubrasolide B and Sinubrasolide E, respectively. The majority of withanolides are predominantly around 70% of absorption, whereas two withanolides showed a lower absorption of less than 60% (Diacetylphiladelphicalactone C and 4β-Hydroxyanomanolide).

#### 2.2.2. BOILED-Egg Prediction

**Figure 5:**
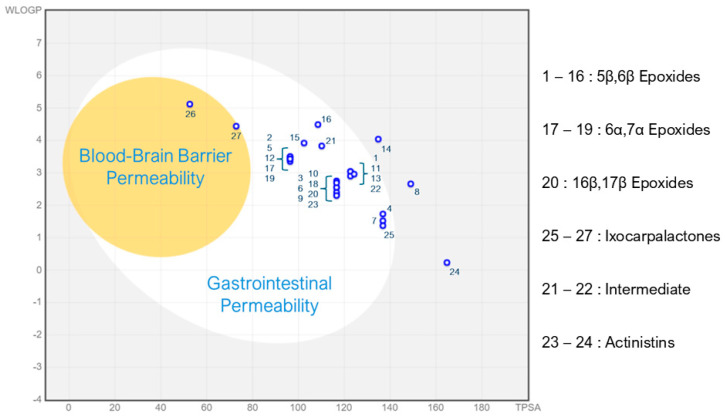
The BOILED-Egg scheme adapted from SwissADME. This plot of TPSA and WLOGP represents two major areas, whereas the withanolides are predicted to cross the gastrointestinal barrier (larger area), the blood–brain (smaller area), or none (outside both areas). Numbers correspond to the chemical structures shown in Figure 1, Figure 2 and Figure 3.

The BOILED-Egg system is a graphic plot (Figure 5), that can predict and show in a visual way, the capability of molecules to surpass both the gastrointestinal barrier and the blood–brain barrier [36]. This model consists of plotting two properties: the Wildman and Crippen LogP (WLogP) and the Topological Polar Surface Area (TPSA) of the chemical structure of the molecules. In a generated image, we can define two zones, one that predicts a good gastrointestinal barrier permeation, and an inside zone that predicts a good blood–brain barrier permeation. These two zones form a shape similar to a boiled egg, therefore its model name.

We have observed, using the BOILED-Egg prediction system, that 25 withanolides would be unable to cross the BBB. However, the two sinubrasolides were plotted inside the BBB permeability area. This suggests an ability for these two ixocarpalactones to cross the BBB, making them potentially harmful for the central nervous system. This fact can be correlated with the higher hydrophobic character of those compounds in comparison to the other withanolides. The Sinubrasolide E structure has only one hydroxyl group, while in Sinubrasolide B, the OH is absent, which limits the hydrogen bond interactions and ionization of the molecule, thus favoring the passage through the BBB.

Also, three of the withanolides would not have good gastrointestinal permeability, whilst the majority would.

#### 2.2.3. AdmetSAR Barrier Predictions

The software admetSAR was able to predict the intestinal barrier and the BBB permeability of withanolides (Table 1; Figure 4). The predicted human intestinal absorption was highly positive for all withanolides, corroborating with the BOILED-Egg and Lipinski’s rule predictions that withanolides could have good intestinal absorption and permeability.

Caco-2 is a cell line, derived from a colorectal adenocarcinoma, commonly utilized as an in vitro model of a human intestinal epithelial barrier for human intestinal absorption studies [37]. The predictions did not show that withanolides could traverse the Caco-2 cells, being strangely opposite to the fact that all predictions for oral permeability and intestinal absorption were positive.

In opposition to the BOILED-Egg, admetSAR predicted that withanolides, in general, have a high chance of surpassing the blood brain barrier, with only six withanolides being predicted as unable to surpass this barrier: Aurelianolide A, Withanolide E, 4β-Hydroxywithanolide E, Physapubenolide, Ixocarpanolide, and 4β-Hydroxyanomanolide. All groups have shown at least 50% probability for accessing the central nervous system (Table 1). This opposite result shows that the different methods of prediction can vary the results, and the real properties should be further investigated using biological systems.

### 2.3. Toxicity Predictions

#### 2.3.1. Hepatotoxicity

Among withanolides analyzed by the admetSAR software, five displayed a probability above the 0.6 tier for hepatotoxicity, being one ixocarpalactone (Sinubrasinolide B), one intermediate withanolide (Withanolide C), the 6a, 7a epoxide Ixocarpanolide, and the 5β,6β epoxides Philadelphicalactone A and 4β-Hydroxyanomanolide. On the other hand, two withanolides were predicted with probability higher than 0.61 to be non-hepatotoxics (Withanolides 14 and 16). The other withanolides displayed probabilities between the 0.51–0.6 tiers (Figure 4), both positively and negatively, with lower variation (Table 2). Chemotherapeutic drugs had a higher score for hepatotoxicity, which agrees with real data for most of these molecules.

The pkCSM prediction tool was used to predict the hepatotoxicity of withanolides. The results, which are shown only as positive or negative, consider the probability of the hepatotoxic effects of withanolides, increasing the robustness of the prediction. This software predicted that 26 of the withanolides are non-hepatotoxic, including those previously predicted as positive by admetSAR (Table 2).

Together, these data suggest that some of the withanolides may be considered safe for liver function, whilst further experimental data is necessary to confirm the possibility of liver tissue damage.

#### 2.3.2. Cardiotoxicity

When analyzed for cardiotoxicity by pkCSM (via interaction with hERG potassium channels), chosen withanolides (Table 2) seem not to interfere in hERG channels, indicating that these molecules are safe for the cardiovascular system.

However, the admetSAR prediction showed a balanced probability between positives and negatives. Eleven withanolides were predicted as non-cardiotoxic, while sixteen were predicted as cardiotoxic. Even though most groups had at least a 0.60 probability to be cardiotoxic, most of the results were all on the borderline between positivity and negativity, thus not being able to define a major profile of cardiotoxicity for the antitumoral withanolides chosen (Table 2).

However, we can associate actinistins to a lower probability of hERG inhibition, with a 0.4366 probability, while intermediate withanolides had the higher probabilities, which could be associated with the absence of epoxide groups (Table 2).

#### 2.3.3. Ames Mutagenesis

Withanolides were predicted to be unable to promote the mutagenicity observed at the Ames mutagenesis test by admetSAR (Table 2; Figure 4), while only one of them was predicted to be mutagenic by pkCSM (Table 2). From this data, we can infer that withanolides are predicted, in a general manner, to be unable to promote mutagenicity.

### 2.4. Metabolism and Excretion Predictions

#### 2.4.1. Cytochrome Interactions

Data obtained using in silico predictions indicate that CYP3A4 is probably the main CYP responsible for withanolide metabolism. Predictions suggest that withanolides do not interact with CYP2D6, another cytochrome responsible for metabolizing xenobiotics. The software also predicted that the majority of withanolides could not inhibit the CYPs available for calculation: CYP3A4, CYP2D6, CYP2C9, CYP2C19, and CYP1A2 (Table 3).

#### 2.4.2. P-Glycoprotein Interaction

Except for four, the pkCSM prediction tool predicted withanolides as P-glycoprotein (P-gp) substrates (Table 1). The admetSAR predictions also corroborated with the positiveness of many compounds, excluding eight that were predicted as non-substrate: all the 6α,7α epoxides, the actinistins, the sinubrasolides, and a single 5β,6β epoxide. The different structures of these groups may deny the P-gp efflux effect.

#### 2.4.3. OCT-2 and MATE-1 Inhibition

Withanolides have shown a homogenous prediction concerning the MATE-1 inhibition. All withanolides were predicted, with high probability, to be unable to inhibit the functioning of the MATE-1 protein. This finding is corroborated by the fact that our system has predicted Imatinib [38] to be a MATE-1 inhibitor (Figure 4). The OCT-2 inhibition was also predicted as negative for the vast majority of withanolides. Withanolide C was the only compound predicted to be an inhibitor of the OCT-2.

#### 2.4.4. BSEP Inhibition

Data obtained from the predictions show a potential of BSEP inhibition by withanolides. A total of 23 of the 27 withanolides were predicted to be BSEP inhibitors; the exceptions were the 5β,6β epoxides Philadelphicalactone C, Withaphysacarpin, Philadelphicalactone A, and Ixocarpalactone A.

### 2.5. Nuclear Receptor Binding

In this work, we have predicted the binding of withanolides to some members of this superfamily: estrogen, androgen, thyroid, glucocorticoid, aromatase, and PPAR-gamma receptors. In a general manner, withanolides are predicted to interact with the NR, with higher frequency to estrogen, androgen, glucocorticoid, aromatase, and PPAR-gamma receptors (Figure 4).

The thyroid receptor binding followed a similar pattern, but with negative binding results for the 16β,17β epoxide Tubocapsanolide E, and the 5β,6β epoxides, compounds **13**, **14**, **15**, and **16**.

### 2.6. Ecotoxicity Predictions

Our findings using the admetSAR predictions (Table 4) showed that withanolides are harmless for honeybees, an important agent in ecological systems. However, all compounds are predicted to be dangerous to aquatic environments, with high fish toxicity and low biodegradability probabilities. Also, the majority of withanolides are predicted to be potentially harmful to Crustacea. The pkCSM tool predicted toxicity concentrations for minnows, which were high, reaching higher than 4 log mM (>10,000 mM). As we have selected potent withanolides, with IC_50_ lower than 10 µM, they can be, in their majority, considered not toxic for minnows. For *Tetrahymena pyriformis* toxicity, the predicted concentrations are also above the potential therapeutic concentrations for withanolides, characterizing them as potentially safe for water environments, according to the pkCSM data.

## 3. Discussion

### 3.1. Absorption and Distribution

Oral drug administration is the preferable non-invasive route for drug delivery to conscious and cooperating patients. However, there are molecules that do not possess physicochemical properties to ensure good bioavailability in the gastrointestinal tract [39].

If a given compound fills in the characteristics predicted in Lipinsky’s rule of five, based in the chemical structure, that molecule may be developed as an oral drug. Every rule has exceptions, and it is no different for Lipinsky’s rule of five. In a general manner, molecules can violate up to one parameter and still be able to have a good prediction. However, Lipinski et al. [34] described some drugs that violate the parameters of the “rule of 5”, such as antibiotics, antifungals, vitamins, and cardiac glycosides, which have pharmaceutical formulations for oral administration, as their chemical structures allow them to act as substrates for natural transporters. In this study we have observed that predicted characteristics of antitumor withanolides fit Lipinsky’s rule of five, suggesting that these molecules may be used as drug development candidates or lead molecules for further development using medicinal chemistry.

It is not uncommon for anticancer drugs to violate two or more of “Lipinski’s rule of 5”; therefore, these drugs may be suitable for the development of intravenous medicines, such as Vincristine, Vinblastine, Doxorubicin, Daunorubicin, and Paclitaxel, among others.

Regarding withanolide compounds, it is observed that many have molecular weights greater than 500 Daltons, due to their complex structure and functional groups. This might make them larger and potentially less likely to meet the molecular weight criterion For example, Aurelianolide A and B, 4β-Hydroxywithanolide E, Physapubenolide, Withanolide C, and Philadelphicalactone C and D, as well as its diacetyl derivative have presented an MW > 500 Da. The n-octanol–water partition coefficient (Kow) is a partition coefficient for the two-phase system consisting of n-octanol and water. Kow serves as a measure of the relationship between lipophilicity and hydrophilicity of a compound. If the Kow value is greater than one, the substance is more soluble in fat-like solvents, such as n-octanol, and less than one if it is more soluble in water. Despite its steroidal scaffold, withanolides often have complex structures with multiple functional groups, including lactones, acetyls, conjugated ketones, hydroxyls, and glycosyls, which give the molecules a greater possibility for carrying out interactions via hydrogen bonds, and thus increasing their hydrophilic character. These features can lead to relatively low LogPow values, which favor some withanolides to meet these criteria. Among the chosen substances in this study, most withanolides presented more than two hydroxyl groups in the structure. The compound 4β-Hydroxyanomanolide is considered to be the most hydrophilic compound endowed by the six OH groups, while Sinubrasolide E and Sinubrasolide B were the most hydrophobic compounds with one and no hydroxyl group, respectively. It is known that the presence of oxygen covalently bonded to hydrogen in the hydroxyl groups (-OH) in the structure of withanolides can act as hydrogen bond donors. Usually, most of the chosen withanolide compounds have hydroxyl groups on C4, C5, C6, C14, C15, and C20. However, one withanolide exceeds the HBD limit of five, the 4β-Hydroxyanomanolide (6-OH groups). Withanolide metabolites are also commonly endowed by several strong electronegative atoms, such as oxygen, that contains a lone pair in functional groups, including hydroxyls, ketones, and esters, that can act as hydrogen bond acceptors. Despite the presence of several hydrogen bond acceptors in the selected compounds, it was not observed that any withanolides exceed the HBA limit of nine, respecting the ten HBA limit criterion.

Overall, due to their complex and often larger structures, many withanolides might not strictly adhere to “Lipinski’s rule of 5”. However, it is important to note that these rules are not absolute predictors of a compound’s bioavailability of drug-likeness. There are many successful drugs that do not meet all these criteria, and exceptions can be made based on additional considerations, such as the specific therapeutic target and mechanism of action. Researchers and drug developers often use Lipinski’s rule of five as a guideline rather than a strict rule, and they consider other factors when assessing the potential of a compound for drug development.

Chemotherapeutics are usually delivered via the parenteral route, with some exceptions, such as the imatinib mesylate, anastrozole, letrozole, and tamoxifen citrate, which were developed as oral drugs. Studies on the ability of withanolides to pass the gastrointestinal barrier may be useful for specific applications and pharmaceutical development. Among the tested withanolides in the BOILED-Egg prediction system, only 4β-Hydroxyanomanolide, Diacetylphiladelphicalactone C, and compound 14 would not be able to pass the gastrointestinal barrier (Figure 5), suggesting that most withanolides would be absorbed by the gastrointestinal system making them optimal candidates for oral administration to patients, an advantage that usually increases the adherence to the treatment.

A very important parameter that can be obtained from the BOILED-Egg plot is the capability of the molecules to surpass the blood–brain barrier and therefore reach the central nervous system. This information will help us to address the possible side effects of these molecules, and to suggest strategies for a better and more specific delivery system/route.

The blood–brain barrier (BBB) is a characteristic feature of the central nervous system blood vessels, that controls the permeability/flux of molecules between the blood and the central nervous system [40], protecting the brain against toxic substances [41]. Therefore, this barrier is extremely important to maintain the cell function and central nervous system homeostasis. Cancer chemotherapy is known to have several side effects, including the cognitive side effects caused by drugs that can cross the blood–brain barrier, affecting homeostasis and cell function, and compromising neuropsychological behavior [42].

Our conflicting findings show that the majority of withanolides are unable to cross the BBB by the BOILED-Egg method, while admetSAR predicted withanolides to be majorly positive. Looking closely at the prediction tools, we can deduct what to take from these results. The BOILED-Egg method shows a high cross-validated accuracy of 0.8775 [36]. While the admetSAR method presents a 0.85 accuracy, it has a 0.66 positive predictive value [43], meaning it has a higher number of false positives, which could be the case for our study, and may account for this discrepancy between models.

Based on the BOILED-Egg method (Figure 5), the majority of withanolides are probably safe for the central nervous system as they do not cross the BBB, reducing the adverse effects of chemotherapy when treating peripheral tumors, such as leukemias. Moreover, delivery strategies must be developed for the treatment of central nervous system tumors with these compounds. The results also suggest that sinubrasolides should be treated separately, as they can access the central nervous system. However, further experimental studies should be carried out to support this suggestion.

Some in silico observations were validated in an ex vivo permeability study [44] that was carried out to investigate the absorption pattern of some withanolides present in *W. somnifera*. A time-dependent absorption of withanolides was observed, including the major withanolides Withaferin A and Withanone, through the intestinal barrier, like BCS class I drugs with high solubility and permeability. In this study, Withanone was detected in plasma [44]. In another study on Withaferin A oral bioavailability in rats [45], it was demonstrated that Withaferin A oral bioavailability was 32.4  ±  4.8% based on intravenous (5 mg/kg) and oral (10 mg/kg) administrations in male rats. According to the authors, the in vitro results showed that Withaferin A could be easily transported across Caco-2 cells and, apparently, it is not a substrate for P-glycoprotein. It was reported that the first-pass metabolism of this withanolide might be the main barrier in achieving good oral bioavailability, since it rapidly decreased, and 27.1% remained within 1 h in a rat intestine–liver in situ perfusion system [45].

### 3.2. Toxicity

The liver is an organ involved in many metabolic pathways and xenobiotic transformations and elimination. However, hepatotoxicity is a common problem observed in antitumor drugs, with many examples of chemotherapics that cause hepatic tissue damage, such as the platinum-based chemical cisplatin, cyclophosphamide, mercaptopurine, and other commonly used drugs. The hepatic damage is often hard to detect and cure [46,47]. Therefore, it is important to find safer antitumoral molecules that cause no hepatic damage or malfunction, that will also be very useful to be introduced as chemotherapeutic agents for patients suffering from hepatic illness or conditions.

AdmetSAR predictions for hepatotoxicity have an accuracy of 0.833 and a sensibility of 0.943, meaning that it is a good tool to discover true hepatotoxic substances, but its low sensitivity of 0.255 can infer many false positives [48]. On the other hand, pkCSM has a low accuracy of 0.658 [49], indicating that the major non-hepatotoxic prediction need to be evaluated further.

The withanolide compounds are known to contain multiple electrophilic groups (two Michael acceptors and an epoxide) in their scaffold, which are associated with adverse drug reactions. The presence of conjugated ketone, lactone, and epoxide groups, generally termed as toxicophores or structural alerts, can often be correlated to some toxicity potential [50]. Found as main metabolites from the medicinal plant *Withania somnifera*, the withanolides, Withaferin A and Withanone, are considered to be the responsible agents for many known pharmacological properties of the Ashwagandha medicinal plant. The hepatoprotective potential has been investigated in animal models using the APAP-induced acute liver injury model. It was suggested that Withaferin A was able to attenuate APAP-induced hepatotoxicity accompanied by hepatic NRF2 activation. The hepatoprotective activity of a crude extract of *W. somnifera* was also found to significantly reduce several elevated hepatotoxicity biomarkers [51]. However, despite the suggested medicinal benefits of *W. somnifera*, recently, several cases of liver toxicity have been reported after the use of commercially available Ashwagandha products. It is suggested that the use of a higher concentrated extract could increase the risk of liver toxicity when compared to the traditional use of Ashwagandha, such as in a tonic, for example. The investigation of the withanolide, Withanone, suggested some toxicophores or structural alerts that are commonly associated with adverse drug reactions. The authors suggested that Withanone can form non-labile adducts with the nucleosides dG, dA, and dC. The Withanone effect can be detoxified by GSH in limited amounts; however, when the GSH system is overwhelmed, severe liver injury occurs, and it can cause DNA damage [52].

Cardiotoxicity is a major issue during preclinical and clinical drug development, a friction factor that leads to failure of lead molecules and projects. An assessment of cardiotoxicity in the early stages of drug development is particularly relevant for drugs that would be used continuously or for an extended period of time in the treatment of chronic diseases or cancer [53]. Cardiotoxicity evaluation and prediction rely on the ionic potassium channels, known as hERG, that are crucial to cardiac function. Molecules that inhibit hERG function can cause severe arrhythmias and sudden death [54]. Doxorubicin and Daunorubicin are representative of the anthracyclines that are known to cause irreversible cardiotoxicity [55], but do not block the hERG channels [56]. Imatinib is a cardiotoxic drug [57], and an hERG inhibitor [58]. In our findings, Imatinib was predicted to inhibit the hERG channels only by admetSAR, while Doxorubicin was not, corroborating to the accuracy of the test.

*Withania somnifera* extracts, which contains Withaferin A, were evaluated in vivo for its cardiotoxic potential. A study has demonstrated that a prior treatment with an extract containing 1.5% of withanolides can attenuate the cardiotoxic effects of Doxorubicin [59]. In another study, extracts could promote cardiotoxicity in neonatal cardiomyocytes, while adult cardiomyocytes remained unharmed by the extracts, providing a cardiotonic effect [60].

The Ames mutagenesis test is a bacterial method, which can be utilized as a pre-clinical method to assess both the mutagenicity and carcinogenicity of compounds [61]. Mutagenesis is an issue that can cause cell damage and possibly evolve into a tumor or fetus malformation. Therefore, many regulatory agencies require data for the mutagenicity and carcinogenicity of drug candidates to proceed in the drug development process [62]. Being developed as a cancer treatment, which is an extended treatment, this data can be utilized in a beneficial way to support safety of withanolide usage.

Concerning the potential of antitumor withanolides for drug development, our findings related to the toxicology of withanolides predict that they are overall relatively safe for major biological systems, including the liver and cardiac function. As a long term antitumoral drug candidate, it also shows low mutagenicity probability. Therefore, withanolides can be promising substances to be included in cancer drug development programs for their safety alongside their good absorption.

### 3.3. Metabolism and Excretion

Withanolides are often metabolized in the liver through enzymatic processes. The liver contains various enzymes, including cytochrome P450 (CYP) enzymes, which play a crucial role in breaking down foreign substances, including drugs and natural compounds. These enzymes help convert withanolides into metabolites that are more water-soluble and can be easily eliminated from the body.

Derived from the phytosterol pathway, withanolides are generally highly oxidized metabolites derived via cycloartenol, which undergo various biochemical transformations, such as hydroxylation, dealkylation, epoxidations, and glycosylation, and are postulated to be catalyzed by cytochrome P450 enzymes (CYP450) [63]. The cytochrome P450 family (CYP) are responsible for the metabolization of approximately 70% of clinical use drugs. CYP3A4 is the main enzyme that accounts for the metabolism of xenobiotics, and is expressed in almost all liver tissue [64]. Because of this role, CYP interaction is one of the mechanisms that undergo drug–drug interactions [65]. Utilizing in silico predictions, we were able to identify possible interactions with CYPs (Table 3).

Recently, a study about the CYP mediated herb–drug interactions of the Ashwagandha medicinal plant, along with case examples of remdesivir, showed that the aqueous extract of Ashwagandha did not show any inhibitory activity towards CYP3A4, CYP2C8, and CYP2D6, and therefore seems to be safe to co-administer with respective substrates [66].

The P-glycoprotein (P-gp) is a transmembrane protein of the ABC (ATP-binding cassette) transporter superfamily. It is decoded by the MDR1 gene and promotes the extrusion of drugs and xenobiotics from cells, granting them resistance to some therapies [67]. We have predicted the susceptibility of withanolides to the effect of these transporters, to see how they would fare in a situation of a multidrug resistant tumor super expressing the MDR1 gene. We used Imatinib and Ibrutinib as positive control drugs, that are known to be P-gp substrates, with a decrease of effectiveness against MDR1 positive tumors [68,69]. The lower effectiveness observed in multidrug resistant tumors can be extrapolated to a withanolide resistance, based on the predictions of P-gp interactions. Supporting this evidence, we have recently demonstrated [18] a higher cytotoxic IC_50_ for Aurelianolide A and B on K562 Lucena-1 leukemia cells that are MDR phenotype due to P-gp increased activity [70]. However, there is in vitro evidence where Withanolide D did not inhibit P-gp activity. Moreover, by blocking the efflux, the cytotoxicity in myeloma cells remained the same [71].

The organic cation transporter 2 (OCT-2) and the multidrug and toxin extrusion protein 1 (MATE-1) are transporters from the ATP-binding cassette (ABC) superfamily. They are abundant in the kidney, controlling the efflux and influx of many molecules, varying from endogenous organic molecules to exogenous drugs and xenobiotics [72]. The interaction with these transporters plays an important role in drug toxicity, drug–drug interactions, and pharmacokinetics [73]. These findings show that withanolides possibly do not interact with the OCT-2 and MATE-1, decreasing the possibility of drug interactions via these kidney transporters.

The bile salt export pump (BSEP) is an ABC transporter localized in the membrane of hepatocytes, transporting bile acids from these cells to the bile. The inhibition of BSEP can lead to a toxic increase of intracellular bile acids, leading to severe liver injury [74]. Albeit the hepatotoxicity data for these findings suggest a possibility of hepatotoxicity from the inhibition of bile acid transporters, and therefore this toxicity should be assessed in future in vitro and in vivo studies.

The nuclear receptor (NR) superfamily are transcription factors that play an important role in biological homeostasis, such as metabolism, reproduction, and inflammation [75]. As a drug candidate, the possible capability of withanolides to bind with nuclear receptors can be a two-edged sword, as this binding can be deleterious by disbalancing homeostasis, causing serious toxic effects. On the other hand, hormone blocking therapies are very valuable to treat hormone responsive tumors, such as prostate and breast cancers, suggesting that withanolides that block hormone receptors may be a very useful tool for the treatment of hormone-sensitive tumors [76,77].

Regarding the in vitro data, there is a study which associates the effects of Withanolide A with glucocorticoid receptors, which are a part of the nuclear receptor superfamily [78]. Alongside our findings, this can indicate the interaction of withanolides with nuclear receptors.

### 3.4. Ecotoxicity

Ecotoxicity refers to the potential harmful effects that chemicals or substances can have on ecosystems and/or the organisms within them. There has been a growing concern for the anthropogenic impacts on the environment, with a rising concern on the impact of drugs and drug wastes in household and industry effluents [79,80]. Therefore, the ecotoxicology of drug candidates can be evaluated to reduce the environmental effects of potentially harmful compounds [81]. Natural bioactive compounds produced by plants or microorganisms can be just as toxic as any anthropogenically-produced chemicals. However, unlike the industrially produced chemicals, such as pesticides, food chemicals, and pharmaceutical chemicals, they are rarely regulated or have their environmental toxicity predicted using QSAR models [82]. The effects of isolated compounds on nature can range from acute toxicity, where high concentrations of a substance lead to immediate harm or death of organisms, to chronic toxicity, where lower concentrations of a substance over a longer period can cause sublethal effects, disrupt reproductive capabilities, and have long-term impacts on the ecosystem’s health. Thus, ecotoxicity studies are conducted to assess the potential risks that substances pose to the environment. The studies involve exposing various organisms, such as aquatic species, plants, and microorganisms, to different concentrations of the substance in question. In this context, a toxicity assessment through computational methods provides knowledge of the chemicals lacking experimental data, and may guide early screening studies for development of safer drugs, and may also serve as an alternative to toxicity studies on animal models [83].

In the current study, all compounds are predicted to have some degree of toxicity in aquatic environments, with high fish toxicity and low biodegradability probabilities. These findings altogether raise an alert and suggest studies on the best production practices and correct waste disposal for withanolides, enlightening scientists and regulators to understand the potential impact of these substances on the environment, guiding decisions on their use and regulation.

## 4. Materials and Methods

### 4.1. Databases

To select and search for the structures and characteristics of withanolides and antineoplastic drugs, we have used databases from the National Institutes of Health (NIH), such as PubMed^®^, the U.S. National Institutes of Health, and PubChem (National Library of Medicine, National Center of Biotechnology Information). To find further information about clinical usage of antineoplastic chemotherapics compounds, searches on DrugBank Online were performed.

### 4.2. Withanolides Selection Criteria

For this work, we have selected 27 withanolides with reported in vitro antitumoral activity with IC_50_ lower than 10 µM [71,84,85,86,87,88,89,90,91,92]. See Supplementary Material Table S1. A plethora of withanolides that represented some of the known types were selected, and allocated by their groups: 5β,6β epoxides (Figure 1), 6α,7α epoxides, 16β,17β epoxides, ixocarpalactones (Figure 2), intermediate withanolides, and actinistins (Figure 3). All withanolides selected were analyzed for in silico predictions in all platforms previously described.

### 4.3. Antineoplastic Drugs

To compare selected antitumoral withanolides to already described molecules and clinical use drugs, we selected 17 widely used antitumor drugs. The selection tried to be representative of drugs with different molecular targets used in clinics for several tumor types; see Supplementary Material Table S2.

### 4.4. ADMET: Physicochemical Descriptors

The structures were designed using the software ACD/ChemSketch 2020.2.0 (ACD/Labs), and the SMILES notations were generated for use in studies; see Supplementary Material Table S1. Each chemical structure from the SMILES notation was submitted for predicting physicochemical and pharmacokinetic properties and toxicity properties using the websites SwissADME (Swiss Institute of Bioinformatics) [93], pkCSM–pharmacokinetics [49], admetSAR v2 [48], and Molinspiration Cheminformatics. All the platforms are free access.

## 5. Conclusions

Withanolides are molecules with good antitumoral potential, being studied as drug candidates worldwide. Considering some limitations, the in silico predictions demonstrated good oral absorption, possibly a low toxicity, a common CYP3A4 metabolization, low inhibition of pharmacokinetics transporters, high probability of nuclear receptor interactions, and a high alert for aquatic environments ecotoxicity.

In summary, the predictions evaluated some positive characteristics that need to be further investigated and confirmed using in vitro and in vivo assays during the drug development process. Our predictions can guide and stimulate the research process for the pharmacological characteristics of withanolides.

## Figures and Tables

**Table 1:** Absorption related properties of withanolides. The parameters were assessed in four prediction tools and compiled in a table. TPSA was calculated using Molinspiration Cheminformatics. SwissADME provided Lipinki’s rule violation and pkCSM predicted the withanolides as yes or no for P-gp substrate. For the admetSAR prediction tool, a probability was obtained, and 0.5 was the threshold between positives and negatives. Values in parenthesis mean the number of withanolides with that characteristic/total number of withanolides. Results are expressed as a mean ± SEM.

	Molinspiration		SwissADME	pkCSM	admetSAR			
Compound Group	TPSA	% Absorption ^a^	Lipinski’s Rule of Five (Violations)	P-gp Substrate	Human Intestinal Absorption	Caco-2 Permeability	Blood–Brain Barrier Permeability	P-gp Substrate
5β,6β epoxides	118.2 ± 3.96	68.23 ± 1.37	0 (7/16); 1 (9/16)	Yes (13/16); No (3/16)	0.9062 ± 0.0115	0.2785 ± 0.0127	0.6125 ± 0.0306	0.5832 ± 0.0108
6α,7α epoxides	103.1 ± 8.74	73.47 ± 2.33	0 (3/3)	Yes (3/3)	0.9391 ± 0.0093	0.3424 ± 0.0128	0.6333 ± 0.0167	0.4871 ± 0.0029
16β,17β epoxide	116.59	68.8	0	Yes	0.8657	0.265	0.5000	0.5123
Intermediate Withanolides	117.2 ± 7.08	68.55 ± 2.45	1 (2/2)	Yes (2/2)	0.9489 ± 0.0204	0.2888 ± 0.0243	0.6250 ± 0.0500	0.5536 ± 0.0098
Actinistins	140.7 ± 24.08	60.50	0 (1/2); 2(1/2)	Yes (2/2)	0.8943 ± 0.0528	0.2268 ± 0.0333	0.5125 ± 0.0876	0.4371 ± 0.0218
Ixocarpalactones	87.42 ± 25.38	78.83 ± 8.75	0 (1/3); 1 (2/3)	Yes (2/3); No (1/3)	0.9471 ± 0.0423	0.3182 ± 0.0602	0.6833 ± 0.0417	0.4561 ± 0.0683

^a^ 109-[0.345xTPSA].

**Table 2:** Toxicity related predictions for withanolides. Cardiotoxicity is represented in the hERG channel inhibition potential and genotoxicity in the Ames mutagenicity test, while hepatotoxicity is represented in a general manner. The pkCSM predicted the withanolides toxicity as yes or no. For the admetSAR prediction tool, a probability was obtained, and 0.5 was the threshold between positives and negatives. Values in parenthesis mean the number of withanolides with that characteristic/total number of withanolides. Results are expressed as a mean ± SEM.

PREDICTION TOOL						
	pkCSM			admetSAR		
Compound Group	Hepatotoxicity	hERG Inhibitor	Ames Mutagenicity	Hepatotoxicity	hERG Inhibition	Ames Mutagenicity
5β,6β epoxides	Yes (1/16); No (15/16)	No (16/16)	Yes (1/16); No (15/16)	0.5295 ± 0.0244	0.6403 ± 0.0250	0.4177 ± 0.0118
6α,7α epoxides	No (3/3)	No (3/3)	No (3/3)	0.5884 ± 0.0252	0.6921 ± 0.0183	0.3131 ± 0.0060
16β,17β epoxide	No	No	No	0.5717	0.5774	0.4100
Intermediate Withanolides	No (2/2)	No (2/2)	No (2/2)	0.6163 ± 0.0661	0.7452 ± 0.0801	0.3447 ± 0.0283
Actinistins	No (2/2)	No (2/2)	No (2/2)	0.5929 ± 0.0021	0.4366 ± 0.0246	0.3089 ± 0.0589
Ixocarpalactones	No (3/3)	No (3/3)	No (3/3)	0.5844 ± 0.0290	0.6722 ± 0.0525	0.2387 ± 0.0759

**Table 3:** Withanolides interactions with cytochrome P450 interactions. Substrate and inhibition probabilities were assessed with the admetSAR prediction tool. Results are expressed as a mean ± SEM. A probability of 0.5 was the threshold between positives and negatives.

admetSAR CYP Predictions								
	Substrate			Inhibition				
Compound Group	CYP3A4 Substrate	CYP2C9 Substrate	CYP2D6 Substrate	CYP3A4 Inhibition	CYP2C9 Inhibition	CYP2C19 Inhibition	CYP2D6 Inhibition	CYP1A2 Inhibition
5β,6β epoxides	0.7375 ± 0.0038	0	0.1026 ± 0.0010	0.1766 ± 0.0173	0.1323 ± 0.0062	0.1032 ± 0.0067	0.0486 ± 0.0012	0.2498 ± 0.0186
6α,7α epoxides	0.7128 ± 0.0108	0	0.0921 ± 0.0005	0.2445 ± 0.0027	0.1190 ± 0.0112	0.1116 ± 0.0019	0.03907 ± 0.0025	0.2613 ± 0.0075
16β,17β epoxide	0.7111	0	0.1016	0.2184	0.1072	0.0935	0.0495	0.1412
Intermediate Withanolides	0.7362 ± 0.0076	0	0.0902 ± 0.0070	0.1314 ± 0.0534	0.1188 ± 0.0145	0.1085 ± 0.0248	0.0661 ± 0.0174	0.3473 ± 0.1894
Actinistins	0.7196 ± 0.0032	0	0.1018 ± 0.0051	0.2853 ± 0.0287	0.1048 ± 0.0129	0.1254 ± 0.0111	0.0372 ± 0.0009	0.1902 ± 0.0195
Ixocarpalactones	0.7485 ± 0.0108	0.0607 ± 0.0607	0.1034 ± 0.0057	0.3099 ± 0.0438	0.0863 ± 0.0304	0.0867 ± 0.0324	0.0456 ± 0.0031	0.2653 ± 0.0607

**Table 4:** Withanolides’ ecotoxicity predictions by the pkCSM and admetSAR tools. Log of toxic concentrations for *Tetrahymena* and minnows are shown in the pkCSM tool. For admetSAR prediction tool, probabilities of toxic effects are evaluated, and 0.5 was the threshold between positives and negatives. Results are expressed as a mean ± SEM.

Prediction of Ecotoxicity						
	pkCSM		admetSAR			
Compound Group	*Tetrahymena pyriformis* Toxicity (log µg/L)	Minnow Toxicity (log mM)	Honey Bee Toxicity	Biodegradation	Crustacea Aquatic Toxicity	Fish Aquatic Toxicity
5β,6β epoxides	0.288 ± 0.002	2.350 ± 0.317	0.2830 ± 0.0150	0.1594 ± 0.0139	0.5281 ± 0.0195	0.9745 ± 0.0024
6α,7α epoxides	0.290 ± 0.004	1.664 ± 0.3495	0.1777 ± 0.0082	0.1917 ± 0.0221	0.5967 ± 0.0433	0.9722 ± 0.0029
16β,17β epoxide	0.285	1.663	0.2019	0.1000	0.4700	0.9730
Intermediate Withanolides	0.290 ± 0.004	1.956 ± 0.5645	0.2219 ± 0.0375	0.1500 ± 0.0250	0.5800 ± 0.0800	0.9906 ± 0.0013
Actinistins	0.285 ± 0.001	3.231 ± 0.3735	0.2507 ± 0.0328	0.2125 ± 0.0125	0.5900 ± 0.0100	0.9767 ± 0.0010
Ixocarpalactones	0.3193 ± 0.024	0.8740 ± 1.226	0.3357 ± 0.0204	0.2167 ± 0.0167	0.5800 ± 0.0577	0.9871 ± 0.0044

## Data Availability

The original contributions presented in this study are included in the article/Supplementary Material. Further inquiries can be directed to the corresponding author(s).

## Supplementary Materials

The following supporting information can be downloaded at: https://www.mdpi.com/article/10.3390/molecules30112457/s1, Table S1: Compound names, Smiles notations used for predictions and references for antitumoral activity; Table S2: Antineoplastic drugs used as standard compounds in predictions and their clinical indication. Table S3: Absorption related properties of withanolides. The parameters were assessed in four prediction tools and compiled in a table. TPSA was calculated using Molinspiration Cheminformatics. SwissADME provided Lipinki’s rule violation and pkCSM predicted the withanolides as yes or no for P-gp substrate. For admetSAR prediction tool, (+) = positive probability, (-) = negative probability. Table S4: Toxicity related predictions for the withanolides. Cardiotoxicity is represented in the hERG channel inhibition potential and genotoxicity in the Ames mutagenicity test, while hepatotoxicity is represented in a general manner. pkCSM predicted the withanolides toxicity as yes or no. For admetSAR prediction tool, (+) = positive probability, (-) = negative probability. Table S5: Withanolides interactions with cytocrome P450 interactions. Subtrate and inhibition probabilities were assessed with the admetSAR prediction tool. (+) = positive probability, (-) = negative probability. Table S6: Withanolides ecotoxicity predictions by the pkCSM and admetSAR tools. Log of toxic concentrations for *Tetrahymena* and minnow are shown in the pkCSM tool. For admetSAR prediction tool, probabilities of toxic effects are evaluated. (+) = positive probability, (-) = negative probability.

## Abbreviations

The following abbreviations are used in this manuscript:

ABC	ATP-binding cassette
ADMET	Absorption, Distribution, Metabolism, Excretion and Toxicity
BBB	Blood–Brain Barrier
BSEP	Bile Salt Export Pump
MATE-1	Multidrug and Toxin Extrusion protein 1
NR	Nuclear Receptors
OCT-2	Organic Cation Transporter 2
P-gp	P-glycoprotein
TPSA	Topological Polar Surface Area
