# Supplementary material for: Anticancer Effects of Withanolides: In Silico Prediction of Pharmacological Properties
Source: Molecules. 2025 Jun 4;30(11):2457. doi: 10.3390/molecules30112457 (PMC12156425; doi:10.3390/molecules30112457)
Supplement: Supplementary file 1 [file molecules-30-02457-s001.zip › molecules-3528861-supplementary.pdf]

Supplementary material for:

Anticancer Effects of Withanolides: In Silico Prediction of Pharmacological Properties

Gustavo Werneck de Souza e Silva [1], André Mesquita Marques [2] and André Luiz Franco Sampaio [1,*]

1 Laboratório de Farmacologia Molecular, Farmanguinhos, Fundação Oswaldo Cruz (FIOCRUZ), Rua Sizenando Nabuco 100, Manguinhos, Rio de Janeiro 21041-250, Brazil; gustavo.werneck@fiocruz.br
2 Laboratório de Tecnologia Para Biodiversidade em Saúde/TecBio, Farmanguinhos, Fundação Oswaldo Cruz (FIOCRUZ), Rua Sizenando Nabuco 100, Manguinhos, Rio de Janeiro 21041-250, Brazil; andre.marques@fiocruz.br
* Correspondence: andre.sampaio@fiocruz.br; Tel.: +55-21-39772443

Table S1. Compound names, Smiles notations used for predictions and references for antitumoral activity.

ID	Name	Smiles notation	Reference
1	Aurelianolide A	CC(=O)O[C@@H]1CC2C3C[C@H]4O[C@]44[C@@H](O)C=CC(=O)[C@]4(C)C3CC[C@]2(C)[C@@]1(O)[C@H](C)C1CC(C)=C(C)C(=O)O1	[18]
2	Withanolide D	CC1=C(C(=O)O[C@H](C1)[C@@](C)([C@H]2CC[C@@H]3[C@@]2(CC[C@H]4[C@H]3C[C@@H]5[C@]6([C@@]4(C(=O)C=C[C@@H]6O)C)O5)C)O)C	[71]
3	Withanolide E	CC1=C(C(=O)O[C@H](C1)[C@@](C)([C@@]2(CC[C@@]3([C@@]2(CC[C@H]4[C@H]3C[C@@H]5[C@]6([C@@]4(C(=O)C=CC6)C)O5)C)O)O)O)C	[84]
4	4β-Hydroxywithanolide E	CC1=C(C(=O)O[C@H](C1)[C@@](C)([C@@]2(CC[C@@]3([C@@]2(CC[C@H]4[C@H]3C[C@@H]5[C@]6([C@@]4(C(=O)C=C[C@@H]6O)C)O5)C)O)O)O)C	[84]
5	Withaferin A	CC1=C(C(=O)O[C@H](C1)[C@@H](C)[C@H]2CC[C@@H]3[C@@]2(CC[C@H]4[C@H]3C[C@@H]5[C@]6([C@@]4(C(=O)C=C[C@@H]6O)C)O5)C)CO	[85]
6	Withalongolide A	CC1=C(C(=O)O[C@H](C1)[C@@H](C)[C@H]2CC[C@@H]3[C@@]2(CC[C@H]4[C@H]3C[C@@H]5[C@]6([C@@]4(C(=O)C=C[C@@H]6O)CO)O5)C)CO	[86]
7	Philadelphicalactone C	C[C@@]1(O)C[C@@H](OC(=O)[C@@H]1C)[C@](C)(O)[C@H]1[C@@H](O)C[C@H]2[C@@H]3C[C@H]4O[C@]44[C@@H](O)C=CC(=O)[C@]4(C)[C@H]3CC[C@]12C	[87]
8	Diacetylphiladelphicalactone C	C[C@@]1(O)C[C@@H](OC(=O)[C@@H]1C)[C@](C)(O)[C@H]1[C@@H](OC(C)=O)C[C@H]2[C@@H]3C[C@H]4O[C@]44[C@H](C=CC(=O)[C@]4(C)[C@H]3CC[C@]12C)OC(C)=O	[87]
9	Withaphysacarpin	C[C@H]1C[C@@H](OC(=O)[C@@H]1C)[C@](C)(O)[C@H]1[C@@H](O)C[C@H]2[C@@H]3C[C@H]4O[C@]44[C@@H](O)C=CC(=O)[C@]4(C)[C@H]3CC[C@]12C	[87]

10	Philadelphicalactone A	C[C@H]1CC(OC(=O)[C@@H]1C)[C@@](C)([C@]2(CC[C@@H]3[C@@]2(CC[C@H]4[C@H]3C[C@@H]5[C@]6([C@@]4(C(=O)C=C[C@@H]6O)C)O5)C)O)O	[87]
11	Physapubenolide	CC=1CC(OC(=O)C=1C)[C@H](C)C1C[C@H](OC(C)=O)C2(O)C3C[C@H]4O[C@]44[C@@H](O)C=CC(=O)[C@]4(C)C3CC[C@]12C	[88]
12	Tubocapsenolide A	CC1=C(C(=O)O[C@H](C1)[C@@H](C)[C@@]2([C@@H](CC3=C2CC[C@H]4[C@H]3C[C@@H]5[C@]6([C@@]4(C(=O)C=C[C@@H]6O)C)O5)O)C)C	[84]
13	Ergosta-2,24-dien-26-oic acid, 27-(acetyloxy)-5,6- epoxy-4,19,22-trihydroxy1-oxo-, 8-lactone, (4ß,5ß,6B, 22R)	CC(=O)OCC1=C(C)CC(OC1=O)[C@@H](C)C1CCC2C3C[C@H]4O[C@]44[C@@H](O)C=CC(=O)[C@]4(CO)C3CC[C@]12C	[11]
14	Ergosta-2,24-dien-26-oic acid, 4,19,27- tris(acetyloxy)-5,6-epoxy22-hydroxy-1-oxo-, 8-lactone, (4B,5B.6B,22R)	CC(=O)OCC1=C(C)CC(OC1=O)[C@@H](C)C1CCC2C3C[C@H]4O[C@]44[C@H](C=CC(=O)[C@]4(COC(C)=O)C3CC[C@]12C)OC(C)=O	[11]
15	Ergosta-2,24-dien-26-oic acid, 4-(acetyloxy)-5,6- epoxy-19,22-dihydroxy-1- oxo-, 8-lactone, (4ß.5B.6$,22R)	CC=1CC(OC(=O)C=1C)[C@@H](C)C1CCC2C3C[C@H]4O[C@]44[C@H](C=CC(=O)[C@]4(CO)C3CC[C@]12C)OC(C)=O	[11]
16	Ergosta-2.24-dien-26-oic acid, 4,19-bis(acetyloxy)- 5,6-epoxy-22-hydroxy-1- oxo-, 8-lactone, (48,5B.6B,22R)	CC=1CC(OC(=O)C=1C)[C@@H](C)C1CCC2C3C[C@H]4O[C@]44[C@H](C=CC(=O)[C@]4(COC(C)=O)C3CC[C@]12C)OC(C)=O	[11]
17	Withanone	CC1=C(C(=O)O[C@H](C1)[C@@H](C)[C@]2(CC[C@@H]3[C@@]2(CC[C@H]4[C@H]3[C@H]5[C@H](O5)[C@@]6([C@@]4(C(=O)C=CC6)C)O)C)O)C	[89]
18	Philadelphicalactone D	C[C@@]1(O)C[C@@H](OC(=O)[C@@H]1C)[C@](C)(O)[C@H]1CC[C@H]2[C@H]3[C@H](CC[C@]12C)[C@@]1(C)C(=O)C=CC[C@]1(O)[C@H]1O[C@@H]31	[87]
19	Ixocarpanolide	C[C@H]1C[C@@H](OC(=O)[C@@H]1C)[C@](C)(O)[C@H]1CC[C@H]2[C@H]3[C@H](CC[C@]12C)[C@@]1(C)C(=O)C=CC[C@]1(O)[C@H]1O[C@@H]31	[87]
20	Tubocapsanolide E	CC=1C[C@@H](OC(=O)C=1C)[C@@H](C)[C@@]12O[C@@H]1C[C@H]1[C@@H]3C[C@H](O)[C@]4(O)[C@@H](O)CCC(=O)[C@]4(C)[C@H]3CC[C@@]12C	[84]
21	Aurelianolide B	CC(=O)O[C@@H]1CC2C3CC=C4[C@@H](O)C=CC(=O)[C@]4(C)C3CC[C@]2(C)[C@@]1(O)[C@H](C)C1CC(C)=C(C)C(=O)O1	[18]
22	Withanolide C	CC1=C(C(=O)O[C@H](C1)[C@@](C)([C@@]2(CC[C@@]3([C@@]2(CC[C@H]4[C@H]3C[C@H]([C@@]5([C@@]4(C(=O)C=CC5)C)Cl)O)C)O)O)O)C	[90]
23	Anomanolide A	C[C@]12CC[C@H]3[C@H]([C@@H]1CC[C@@]2(C4C[C@@]5(C[C@H]4OC(=O)[C@]5(C)O)C)O)C[C@@H]6[C@]7([C@@]3(C(=O)C=C[C@@H]7O)C)O6	[84]
24	4β-Hydroxyanomanolide	O[C@H]1C=CC(=O)[C@]2(C)[C@H]3CC[C@@]4(C)[C@@H](C[C@@H](O)[C@]4(O)[C@@H]4C[C@]5(C)C[C@H]4OC(=O)[C@]5(C)O)[C@@H]3C[C@H](O)[C@]12O	[91]

25	Ixocarpalactone A	C[C@@H]1[C@@H](C)C(=O)OC1[C@H](O)[C@](C)(O)[C@H]1[C@@H](O)C[C@H]2[C@@H]3C[C@H]4O[C@]44[C@@H](O)C=CC(=O)[C@]4(C)[C@H]3CC[C@]12C	[87]
26	Sinubrasolide B	C[C@H]1[C@H]2[C@H](C[C@@H]3[C@@]2(CC[C@H]4[C@H]3CCC5=CC(=O)C=C[C@]45C)C)O[C@@H]1C6[C@@H]([C@H](C(=O)O6)C)C	[92]
27	Sinubrasolide E	O=C1C=C2CC[C@@H]3[C@H](CC[C@]4(C)[C@@H]5[C@H](C[C@@H]34)O[C@]3(OC(=O)[C@@H](C)[C@H]3C)[C@@H](O)[C@H]5C)[C@@]2(C)C=C1	[92]

Table S2. Antineoplastic drugs used as standard compounds in predictions and their clinical indication.

**Antineoplastic**	**Clinical indication**
Staurosporine	Kinase inhibitor.
Imatinib	Inhibitor of BCL-ABL kinase in chronic myeloid leukemia.
Vincristine	Vinca alkaloid used against many tumor types: leukemias, lymphomas, neuroblastomas, rhabdomyosarcomas.
Vinblastine	Vinca alkaloid with a wide tumor type effectiveness: lymphomas, neuroblastomas, breast and testicular cancer.
Doxorubicin	Anthracycline used in the treatment of many tumor types.
Cyclophosphamide	Pro-drug used in the treatment of lymphomas and leukemias.
Mercaptopurine	Inhibits purine metabolism, often used against leukemias.
Cytarabine	Inhibits the DNA synthesis, specially used against acute myeloid leukemias.

Binimetinib	MEK1/2 inhibitor, used to treat metastatic melanomas.
Letrozole	Aromatase inhibitor, used in the treatment of hormone positive breast cancer.
Ibrutinib	Burton’s tyrosine inhibitor, effective against lymphomas and leukemias.
Daunorubicin	Anthracycline often used in combination with Cytarabine in the treatment of acute myeloid leukemias.
Bortezomib	Used in the treatment of mantle cell lymphomas and myelomas.
Gefitinib	Used to treat metastatic or advanced non-small cell lung cancer.
Paclitaxel	Microtubule inhibitor, effective against sarcomas, breast, lung and ovarian cancer, including metastatic tumors.
Apalutamide	Androgen receptor antagonist, effective against prostate cancer.
Cisplatin	Platinum based molecule, used in the treatment of ovarian, bladder and testicular tumors.

Table S3. Absorption related properties of withanolides. The parameters were assessed in four prediction tools and compiled in a table. TPSA was calculated using Molinspiration Cheminformatics. SwissADME provided Lipinki's rule violation and pkCSM predicted the withanolides as yes or no for P-gp substrate. For admetSAR prediction tool, (+) = positive probability, (-) = negative probability.

	**Molinspiration**		**SwissADME**	**pkCSM**	**admetSAR**			
**Compound ID**	**TPSA**	**% Absorption [a]**	**Lipinski's Rule of Five (Violations)**	**P-gp Substrate**	**Human Intestinal Absorption**	**Caco-2 Permeability**	**Blood-Brain Barrier Permeability**	**P-gp Substrate**
1	122.67	66.7	1	Yes	0.9472 (+)	0.7566 (-)	0.5 (-)	0.5662 (+)
2	96.36	75.8	0	Yes	0.9485 (+)	0.6274 (-)	0.5250 (+)	0.5144 (-)
3	116.59	68.8	0	Yes	0.8604 (+)	0.6455 (-)	0.5250 (-)	0.6452 (+)
4	136.82	61.8	1	Yes	0.8604 (+)	0.7133 (-)	0.5250 (-)	0.5949 (+)
5	96.36	75.8	0	Yes	0.8983 (+)	0.6673 (-)	0.6250 (+)	0.5948 (+)
6	116.59	68.8	0	Yes	0.8498 (+)	0.7411 (-)	0.6250 (+)	0.5949 (+)
7	136.82	61.8	1	Yes	0.8656 (+)	0.7700 (-)	0.6000 (+)	0.6092 (+)
8	148.97	57.6	1	Yes	0.9290 (+)	0.7926 (-)	0.6000 (+)	0.6178 (+)
9	116.59	68.8	0	Yes	0.8656 (+)	0.7344 (-)	0.6000 (+)	0.5469 (+)
10	116.59	68.8	0	Yes	0.8746 (+)	0.7083 (-)	0.5250 (+)	0.5096 (+)
11	122.67	66.7	1	No	0.9472 (+)	0.7376 (-)	0.5000 (-)	0.5984 (+)
12	96.36	75.8	0	Yes	0.9568 (+)	0.6456 (-)	0.7000 (+)	0.5485 (+)
13	122.67	66.7	1	Yes	0.8419 (+)	0.7505 (-)	0.6000 (+)	0.6136 (+)
14	134.82	62.5	1	No	0.9585 (+)	0.7891 (-)	0.8750 (+)	0.6347 (+)
15	102.44	73.7	1	Yes	0.9219 (+)	0.7303 (-)	0.7250 (+)	0.5719 (+)
16	108.51	71.6	1	No	0.9733 (+)	0.7349 (-)	0.8500 (+)	0.5989(+)
17	96.36	75.8	0	Yes	0.9576 (+)	0.6472 (-)	0.6000 (+)	0.5098 (-)
18	116.59	68.8	0	Yes	0.9298 (+)	0.6830 (-)	0.6500 (+)	0.5102 (-)
19	96.36	75.8	0	Yes	0.9298 (+)	0.6427 (-)	0.6500 (+)	0.5186 (-)
20	116.59	68.8	0	Yes	0.8657 (+)	0.7350 (-)	0.5000 (-)	0.5123 (+)

21	110.14	71.0	1	Yes	0.9692 (+)	0.7355 (-)	0.5750 (+)	0.5438 (+)
22	124.29	66.1	1	Yes	0.9285 (+)	0.6870 (-)	0.6750 (+)	0.5633 (+)
23	116.59	68.8	0	Yes	0.9471 (+)	0.7400 (-)	0.6000 (+)	0.5847 (-)
24	164.74	52.2	2	Yes	0.8415 (+)	0.8065 (-)	0.5750 (-)	0.5412 (-)
25	136.82	61.8	1	Yes	0.8627 (+)	0.7992 (-)	0.6000 (+)	0.5897 (+)
26	52.61	90.8	1	No	0.9948 (+)	0.6002 (-)	0.7250 (+)	0.6355 (-)
27	72.84	83.9	0	Yes	0.9837 (+)	0.6459 (-)	0.7250 (+)	0.5858 (-)

[a] 109-[0.345xTPSA].

Table S4. Toxicity related predictions for the withanolides. Cardiotoxicity is represented in the hERG channel inhibition potential and genotoxicity in the Ames mutagenicity test, while hepatotoxicity is represented in a general manner. pkCSM predicted the withanolides toxicity as yes or no. For admetSAR prediction tool, (+) = positive probability, (-) = negative probability.

PREDICTION TOOL						
	pkCSM			admetSAR		
**COMPOUND ID**	**Hepatotoxicity**	**hERG Inhibitor**	**Ames Mutagenicity**	**Hepatotoxicity**	**hERG Inhibition**	**Ames Mutagenicity**
1	No	No	No	0.5034 (-)	0.3937 (-)	0.5600 (-)
2	No	No	No	0.6033 (+)	0.6831 (+)	0.5800 (-)
3	No	No	No	0.6068 (+)	0.8123 (+)	0.6400 (-)
4	No	No	No	0.6232 (+)	0.7005 (+)	0.6500 (-)
5	No	No	No	0.5655 (+)	0.6673 (+)	0.5102 (-)
6	Yes	No	No	0.5342 (+)	0.4061 (-)	0.5548 (-)
7	No	No	Yes	0.5140 (+)	0.3644 (-)	0.6000 (-)
8	No	No	No	0.5756 (-)	0.4203 (-)	0.6500 (-)
9	No	No	No	0.6066 (+)	0.6451 (+)	0.5900 (-)
10	No	No	No	0.7029 (+)	0.6583 (+)	0.6400 (-)
11	No	No	No	0.5607(+)	0.6461 (+)	0.5170 (-)
12	No	No	No	0.5961 (+)	0.4016 (-)	0.6154 (-)
13	No	No	No	0.5105 (-)	0.6496 (-)	0.5348 (-)
14	No	No	No	0.6405 (-)	0.3986 (-)	0.5348 (-)
15	No	No	No	0.5835(-)	0.7022 (+)	0.5848(-)
16	No	No	No	0.6280 (-)	0.7636 (+)	0.5548 (-)
17	No	No	No	0.5858 (+)	0.7284 (+)	0.6753 (-)
18	No	No	No	0.5461 (+)	0.6772 (+)	0.6953 (-)
19	No	No	No	0.6333 (+)	0.6706 (+)	0.6900 (-)
20	No	No	No	0.5717 (+)	0.4226 (-)	0.5900 (-)
21	No	No	No	0.5502 (+)	0.6651 (+)	0.6270 (-)
22	No	No	No	0.6824 (+)	0.8253 (+)	0.6836 (-)
23	No	No	No	0.5950 (+)	0.5392 (-)	0.7500 (-)
24	No	No	No	0.5908 (+)	0.5877 (-)	0.6323 (-)
25	No	No	No	0.5783 (+)	0.4055 (-)	0.6100 (-)
26	No	No	No	0.6375 (+)	0.7722 (+)	0.8270 (-)
27	No	No	No	0.5375 (+)	0.6500 (+)	0.8470 (-)

Table S5. Withanolides interactions with cytocrome P450 interactions. Subtrate and inhibition probabilities were assessed with the admetSAR prediction tool. (+) = positive probability, (-) = negative probability.

admetSAR CYP predictions

	Substrate			Inhibition				
**Compound ID**	**CYP3A4 substrate**	**CYP2C9 substrate**	**CYP2D6 substrate**	**CYP3A4 inhibition**	**CYP2C9 inhibition**	**CYP2C19 inhibition**	**CYP2D6 inhibition**	**CYP1A2 inhibition**
1	0.7425 (+)	10000 (-)	0.9020 (-)	0.8569 (-)	0.8226 (-)	0.8734 (-)	0.9491 (-)	0.6090 (-)
2	0.7523 (+)	1.0000 (-)	0.8956 (-)	0.8598 (-)	0.8654 (-)	0.8991 (-)	0.9546 (-)	0.6315 (-)
3	0.7134 (+)	1.0000 (-)	0.8958 (-)	0.7750 (-)	0.8408 (-)	0.8738 (-)	0.9484 (-)	0.8267 (-)
4	0.7206 (+)	1.0000 (-)	0.8924 (-)	0.7750 (-)	0.8408 (-)	0.8738 (-)	0.9484 (-)	0.8267 (-)
5	0.7332 (+)	1.0000 (-)	0.8921 (-)	0.8547 (-)	0.8867 (-)	0.9390 (-)	0.9517 (-)	0.7829 (-)
6	0.7382 (+)	1.0000 (-)	0.8921 (-)	0.8840 (-)	0.8986 (-)	0.9229 (-)	0.9499 (-)	0.8151 (-)
7	0.7367 (+)	1.0000 (-)	0.8985 (-)	0.7300 (-)	0.8816 (-)	0.8936 (-)	0.9576 (-)	0.7898 (-)
8	0.7552 (+)	1.0000 (-)	0.9043 (-)	0.7504 (-)	0.8678 (-)	0.8602 (-)	0.9584 (-)	0.7308 (-)
9	0.7326 (+)	1.0000 (-)	0.8985 (-)	0.7300 (-)	0.8816 (-)	0.8936 (-)	0.9576 (-)	0.7898 (-)
10	0.7205 (+)	1.0000 (-)	0.8952 (-)	0.7243 (-)	0.8828 (-)	0.8569 (-)	0.9612 (-)	0.7485 (-)
11	0.7383 (+)	1.0000 (-)	0.9020 (-)	0.8569 (-)	0.8226 (-)	0.8734 (-)	0.9491 (-)	0.6090 (-)
12	0.7086 (+)	1.0000 (-)	0.8911 (-)	0.7905 (-)	0.8941 (-)	0.9371 (-)	0.9510 (-)	0.7030 (-)
13	0.7548 (+)	1.0000 (-)	0.8986 (-)	0.8893 (-)	0.8744 (-)	0.9111 (-)	0.9469 (-)	0.8013 (-)
14	0.7511 (+)	1.0000 (-)	0.9003 (-)	0.9316 (-)	0.8957 (-)	0.8949(-)	0.9456 (-)	0.8057 (-)
15	0.7525 (+)	1.0000 (-)	0.9004(-)	0.8505 (-)	0.8507 (-)	0.9245 (-)	0.9479 (-)	0.7603 (-)
16	0.7500 (+)	1.0000 (-)	0.9003 (-)	0.9148 (-)	0.8765 (-)	0.9219 (-)	0.9455 (-)	0.7726 (-)
17	0.6913 (+)	1.0000 (-)	0.9069 (-)	0.7609 (-)	0.8586 (-)	0.8921 (-)	0.9560 (-)	0.7538 (-)
18	0.7210 (+)	1.0000 (-)	0.9084 (-)	0.7528 (-)	0.8922 (-)	0.8865 (-)	0.9634 (-)	0.7312 (-)
19	0.7260 (+)	1.0000 (-)	0.9084 (-)	0.7528 (-)	0.8922 (-)	0.8865 (-)	0.9634 (-)	0.7312 (-)
20	0.7111 (+)	1.0000 (-)	0.8984 (-)	0.7816 (-)	0.8928 (-)	0.9065 (-)	0.9505 (-)	0.8588 (-)
21	0.7437 (+)	1.0000 (-)	0.9168 (-)	0.8153 (-)	0.8957 (-)	0.9163 (-)	0.9513 (-)	0.5366 (+)
22	0.7286 (+)	1.0000 (-)	0.9028 (-)	0.9220 (-)	0.8668 (-)	0.8667 (-)	0.9165 (-)	0.8421 (-)
23	0.7230 (+)	1.0000 (-)	0.8931 (-)	0.6861 (-)	0.8823 (-)	0.8857 (-)	0.9620 (-)	0.7903 (-)
24	0.7166 (+)	1.0000 (-)	0.9033 (-)	0.7434 (-)	0.9081 (-)	0.8635 (-)	0.9637 (-)	0.8293 (-)
25	0.7296 (+)	1.0000 (-)	0.8952 (-)	0.6270 (-)	0.8545 (-)	0.8607 (-)	0.9500 (-)	0.7636 (-)
26	0.7488 (+)	1.0000 (-)	0.9071 (-)	0.6690 (-)	0.9310 (-)	0.9068 (-)	0.9530 (-)	0.7877 (-)
27	0.7671 (+)	0.8178 (-)	0.8875 (-)	0.7742 (-)	0.9556 (-)	0.9725 (-)	0.9603 (-)	0.6527 (-)

Table S6. Withanolides ecotoxicity predictions by the pkCSM and admetSAR tools. Log of toxic concentrations for *Tetrahymena* and minnow are shown in the pkCSM tool. For admetSAR prediction tool, probabilities of toxic effects are evaluated. (+) = positive probability, (-) = negative probability.

Prediction of Ecotoxicity						
	**pkCSM**		**admetSAR**			
**COMPOUND ID**	***Tetrahymena pyriformis* toxicity (log µg/L)**	**Minnow Toxicity (log mM)**	**Honey bee toxicity**	**Biodegradation**	**Crustacea aquatic toxicity**	**Fish aquatic toxicity**
1	0.286	2.541	0.6874 (-)	0.8750 (-)	0.5400 (-)	0.9871 (+)
2	0.305	1.178	0.7946 (-)	0.8250 (-)	0.6400 (+)	0.9852 (+)
3	0.286	2.578	0.8353 (-)	0.9250 (-)	0.6500 (+)	0.9759 (+)
4	0.285	3.794	0.8040 (-)	0.9250 (-)	0.6300 (+)	0.9759 (+)
5	0.299	0.738	0.7451 (-)	0.8750 (-)	0.5500 (-)	0.9671 (+)
6	0.287	1.426	0.7191 (-)	0.8500 (-)	0.5400 (-)	0.9595 (+)
7	0.285	4.26	0.6595 (-)	0.8500 (-)	0.5800 (+)	0.9622 (+)
8	0.285	4.852	0.6197 (-)	0.7750 (-)	0.6200 (+)	0.9732 (+)
9	0.285	3.003	0.6721 (-)	0.8250 (-)	0.5700 (+)	0.9622 (+)
10	0.288	3.012	0.7681 (-)	0.8000 (-)	0.5700 (+)	0.9727 (+)
11	0.285	2.992	0.7079 (-)	0.8750 (-)	0.5100 (-)	0.9871 (+)
12	0.296	0.971	0.7358 (-)	0.9000 (-)	0.5000 (-)	0.9772 (+)
13	0.285	0.564	0.6624(-)	0.7250 (-)	0.5200 (-)	0.9647 (+)
14	0.285	1.624	0.6577 (-)	0.8250 (-)	0.5300 (-)	0.9849 (+)
15	0.286	1.776	0.7068 (-)	0.7750(-)	0.5700 (-)	0.9700 (+)
16	0.285	2.29	0.6962 (-)	0.8250 (-)	0.5500 (-)	0.9878 (+)
17	0.299	1.322	0.8063 (-)	0.8500 (-)	0.5100 (+)	0.9780 (+)
18	0.285	2.363	0.8335 (-)	0.8000 (-)	0.6400 (+)	0.9693 (+)
19	0.287	1.307	0.8270 (-)	0.7750 (-)	0.6400 (+)	0.9693 (+)
20	0.285	1.663	0.7981 (-)	0.9000 (-)	0.5300 (-)	0.9730 (+)
21	0.293	1.391	0.7407 (-)	0.8250 (-)	0.5000 (-)	0.9893 (+)
22	0.286	2.52	0.8156 (-)	0.8750 (-)	0.6600 (+)	0.9918 (+)
23	0.286	2.857	0.7166 (-)	0.7750 (-)	0.5800 (+)	0.9757 (+)
24	0.285	3.604	0.7821 (-)	0.8000 (-)	0.6000 (+)	0.9777 (+)
25	0.285	3.295	0.6491 (-)	0.7500 (-)	0.5200 (-)	0.9818 (+)
26	0.365	-0.676	0.7046 (-)	0.8000 (-)	0.6800 (+)	0.9959 (+)
27	0.308	0.003	0.6391 (-)	0.8000 (-)	0.5800 (+)	0.9835 (+)
